# The FOCUS trial: cognitive remediation plus standard treatment versus standard treatment for patients at ultra-high risk for psychosis: study protocol for a randomised controlled trial

**DOI:** 10.1186/s13063-014-0542-8

**Published:** 2015-01-27

**Authors:** Louise B Glenthøj, Birgitte Fagerlund, Lasse Randers, Carsten R Hjorthøj, Christina Wenneberg, Kristine Krakauer, Astrid Vosgerau, Christian Gluud, Alice Medalia, David L Roberts, Merete Nordentoft

**Affiliations:** Mental Health Centre Copenhagen, Copenhagen University Hospital, DK-2400 Copenhagen, Denmark; Centre for Clinical Intervention and Neuropsychiatric Schizophrenia Research, CINS, DK-2600 Glostrup, Denmark; Centre for Neuropsychiatric Schizophrenia Research (CNSR), Mental Health Centre Glostrup, Copenhagen University Hospital, DK-2600 Glostrup, Denmark; Centre for Rehabilitation for Brain Injury, DK-2300 Copenhagen, Denmark; Copenhagen Trial Unit, Centre for Clinical Intervention Research, Department 7812, Rigshospitalet, Copenhagen University Hospital, DK-2100 Copenhagen, Denmark; Columbia University Medical Center, New York, NY 10032 USA; Department of Psychiatry, Division of Schizophrenia and Related Disorders, University of Texas Health Science Center, San Antonio, TX 78229 USA

**Keywords:** At-risk mental state, Ultra-high risk for psychosis, Prodromal schizophrenia, Prodromal intervention, Cognitive remediation, Cognitive training, Social cognitive training, Neurocognitive training

## Abstract

**Background:**

Cognitive deficits are a distinct feature among people at ultra-high risk (UHR) for psychosis and pose a barrier to functional recovery. Insufficient evidence exists on how to ameliorate these cognitive deficits in patients at UHR for psychosis and hence improve daily living and quality of life. The aim of the trial is to investigate whether cognitive remediation can improve cognitive and psychosocial function in patients at UHR for psychosis.

**Methods:**

The FOCUS trial (Function and Overall Cognition in Ultra-high risk States) is a randomised, parallel group, observer-blinded clinical trial enrolling 126 patients meeting the standardised criteria of being at UHR for psychosis. Patients are recruited from psychiatric in- and outpatient facilities in the Copenhagen catchment area. Patients are randomised to one of the two treatment arms: cognitive remediation plus standard treatment versus standard treatment. The cognitive remediation consists of 24 weekly group-based and manualised sessions targeting neurocognition and social cognition. In addition to the group sessions, the patients will be offered 12 individual sessions aiming at maximising the transfer of the effects of the cognitive training to their everyday lives. Follow-up assessments will be conducted at 6 and 12 months after randomisation. The primary outcome is the composite score on the Brief Assessment of Cognition in Schizophrenia at cessation of treatment after 6 months. Secondary outcomes are social and daily functioning, psychosis-like symptoms, negative symptomatology, and depressive symptomatology as measured with the Personal and Social Performance Scale, Brief Psychiatric Rating Scale-Expanded Version, Scale for the Assessment of Negative Symptoms, and the Montgomery-Åsberg Depression Rating Scale.

**Discussion:**

This is the first trial to evaluate the effects of neurocognitive and social cognitive remediation in UHR patients. The FOCUS trial results will provide evidence on the effect of targeted and comprehensive cognitive rehabilitation on cognition, daily living, and symptomatology as well as long-term outcome in preventing transition to psychosis in UHR patients.

**Trial registration:**

ClinicalTrials.gov NCT 02098408. Date of registration 18 March 2014.

## Background

### The prodromal phase of schizophrenia and other psychotic disorders

Schizophrenia is a debilitating disorder with a point prevalence of 0.6% [1]. The construct of the ultra-high-risk (UHR) state for schizophrenia and other psychotic disorders captures the putative prodromal phase of psychosis, which is seen as a forerunner of frank psychosis. Patients in this UHR state present discrete yet identifiable psychotic symptoms. Intervention studies targeting the UHR state for psychosis have increased during the last decade [2]. Potentially, such interventions could avoid, ameliorate, or delay progression to psychosis. Furthermore, initiating appropriate treatment as early as possible has the potential to improve both the clinical and functional outcomes of patients.

The most recent meta-analysis on prodromal intervention in psychosis assessed 10 randomised clinical trials aiming at preventing psychosis [3]. The intervention strategies in these trials encompass antipsychotic medication, omega-3 fatty acids, psychosocial, and cognitive behavioural interventions and integrative therapy [4-13]. The results from these trials are promising, but evidence is still too scarce to be conclusive. None of the studies on prodromal intervention have explored the potential effects of extensive cognitive remediation on improving cognitive and psychosocial functioning in prodromal patients or the potential to prevent transition to psychosis.

### Cognitive dysfunctions and cognitive remediation in schizophrenia

It is well known that impairments in cognition are characteristic and pervasive in schizophrenia and significantly influence the functional outcome [14-16]. The cognitive and the associated functional impairments cause patients with schizophrenia to experience disturbances in areas such as independent living, social relationships, and educational attainment [15,17] and are a strong predictor of response to psychiatric rehabilitation [18].

Cognition encompasses neurocognitive and social cognitive processes, which are regarded as two distinct but interrelated domains [19]. Neurocognition can be defined as “processes of linking and appraising information. It includes abilities like speed of processing, attention, verbal and visual learning and memory, working memory as well as reasoning and problem solving” [20], whereas social cognition can be defined as “the mental operations that underlie social interactions, including perceiving, interpreting, and generating responses to the intentions, dispositions, and behaviours of others” [21]. It is hypothesised that social cognition acts as a mediator between neurocognition and functional outcome. Evidence for this hypothesis has been found in several studies [20,22-27]. These findings imply that social cognition is proximal to the patients’ everyday functioning. In this context social cognition can be seen as critical for the daily functioning of the patients (e.g., community functioning, interpersonal relationships, and ultimately quality of life) [28]. Building on this rationale we decided to target both neurocognition and social cognition in our trial.

A promising method to alleviate cognitive deficits is using cognitive remediation (CR). It can be defined as “a behavioural training based intervention that aims to improve cognitive processes (attention, memory, executive function, social cognition or metacognition) with the goal of durability and generalization” [29]. In the most recent meta-analysis of CR in schizophrenia, Wykes et al. reviewed 40 trials. The effect of CR on cognition, functioning, and symptoms was assessed post-treatment and at follow-up. They demonstrated a significant positive effect in most cognitive domains [global cognition effect size 0.45 with 95% confidence interval (CI) = 0.31-0.59] and on functional outcomes (effect size 0.42 with 95% CI = 0.22-0.62). The effect appears to be durable (effect size 0.43 with 95% CI = 0.18-0.67). It is noted that the impact of CR on functional outcomes was significantly greater in studies also providing psychiatric rehabilitation [29]. This leads to the conclusion that CR seems an important and beneficial target of intervention in people with schizophrenia. Research indicates that CR is also effective in other severe mental illnesses [30,31].

### Cognitive dysfunctions and cognitive remediation among people at ultra-high risk for psychosis

A clinical staging model of the cognitive deficits in schizophrenia has been proposed. It suggests that there is a cognitive decline between early stages of the illness, implying that the cognitive deficits become increasingly severe as the illness develops [32]. Most studies favour a neurodevelopmental model suggesting that the cognitive deficits are already established before illness onset and remain mostly stable during the illness [33,34].

There is an increasing body of evidence demonstrating that patients in the UHR state show significant deficits in multiple cognitive domains [35-38]. In their meta-analysis on cognitive dysfunctions in the UHR state, Fusar-Poli et al. found cognitive deficits associated with the UHR state in attention, verbal fluency, visual and verbal memory, working memory, and executive functioning [35]. An area of cognition that also has been found to be impaired in the UHR state is social cognition, which appears to be closely related to functional outcome [22,39]. Evidence shows that the cognitive deficits in UHR patients have a significant impact on their level of functioning [37,40], an even greater impact than symptom severity [22,37].

Reviewing the literature in the electronic databases (PubMed, EMBASE, Clinical Trials.gov, and WHO Trial Portal) for studies related to the terms “cognitive remediation, prodromal psychosis, or ultra-high risk states” resulted in only two published studies that have examined the effect of CR on UHR patients. Bechdolf et al. offered CR as part of a broader integrated intervention programme [9]. They found a beneficial effect of the integrated intervention, but it was not possible to isolate the effect of the CR. A pilot study by Rauchensteiner et al. compared the effectiveness of CR in 10 UHR patients to that of CR in 16 patients with schizophrenia and found relatively greater improvements in cognitive functioning (improved long-term memory functions as well as attention) in the former group [41]. However, as the authors also state, this finding needs to be replicated in studies using larger sample sizes. Furthermore, the observational design generally hinders fair assessment of the benefits of interventions [42]. Of note, the two studies included short-term remediation programmes (10-12 sessions) without direct assessment of the effect on daily functioning. Accordingly, there is a need for randomised clinical trials that apply more extensive cognitive training on larger UHR samples.

The aim of the FOCUS trial is to investigate to what extent CR may improve cognitive abilities and the associated psychosocial function in patients at UHR for psychosis. Bearing in mind the disabling consequences of cognitive deficits in schizophrenia and psychosis-like states, it seems vital to target these deficits to improve the everyday functioning of the patients. Knowing that the cognitive deficits manifest themselves in the UHR state, we expect that cognitive deficits may be even more amenable to treatment at this early stage of illness than what has previously been found at a more chronic stage [43,44]. Accordingly, targeting cognitive dysfunctions in the prodromal phase of psychosis may be the optimal time to intervene.

If a beneficial effect of CR on the cognitive and psychosocial dysfunctions in UHR patients is found, this would point to future randomised clinical trials and later potential implementation of CR in facilities offering early intervention in psychosis in order to enhance the ability of patients to function in their daily life.

### Hypotheses

In the present study we will examine whether:

1. Cognitive remediation therapy will be superior to standard treatment in improving cognitive functioning in UHR patients (null hypothesis: no difference between the two intervention groups).
2. Cognitive remediation therapy will be superior to standard treatment in improving psychosocial functioning and clinical symptoms in UHR patients (null hypothesis: no difference between the two intervention groups).

## Methods

### Recruitment

**Figure 1:**
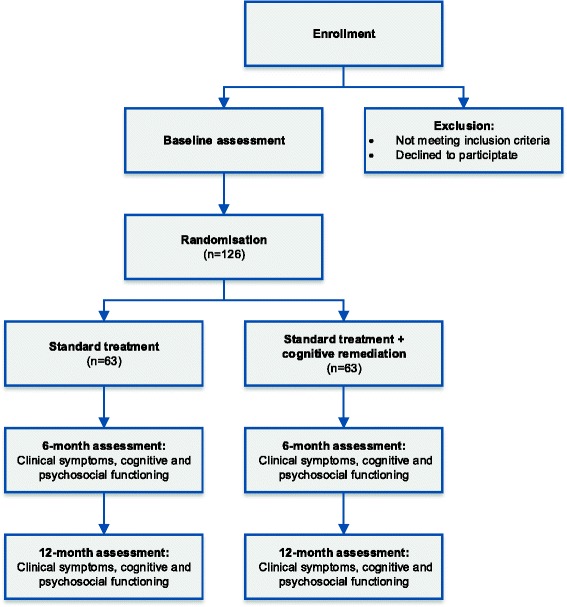
**Flowchart of the FOCUS trial.**

The FOCUS trial is a randomised, blinded, parallel-group superiority clinical trial, enrolling a total of 126 help-seeking patients from in- and outpatient facilities in the catchment area of Copenhagen (Figure 1). Patients meeting standardised ‘at-risk’ criteria based on the Comprehensive Assessment of At-Risk Mental States (CAARMS) [45] will be approached about participating in this clinical trial. Patients will be randomised (1:1) to intensive CR plus standard treatment versus standard treatment, as described in further detail in the section Interventions.

### Inclusion criteria

Participants aged 18-40 years who provide written informed consent and fulfil criteria for being at UHR for psychosis (defined by one or more of the following):

- **Vulnerability (Trait and State Risk Factor) Group:** Individuals with a combination of a trait risk factor (schizotypal personality disorder or a family history of psychotic disorder in a first degree relative) and a significant deterioration in functioning or sustained low functioning during the past year.
- **Attenuated Psychotic Symptoms (APS) Group:** Individuals with sub-threshold (intensity or frequency) positive psychotic symptoms. The symptoms must have been present during the past year.
- **Brief Limited Intermittent Psychotic Symptoms (BLIPS) Group:** Individuals with a recent history of frank psychotic symptoms that resolved spontaneously (without antipsychotic medication) within 1 week. The symptoms must have been present during the past year.

### Exclusion criteria

Exclusion criteria are: a past history of a treated or untreated psychotic episode of 1 week’s duration or longer, psychiatric symptoms that are explained by a physical illness with psychotropic effect or acute intoxication (e.g., cannabis use), a diagnosis of a serious developmental disorder (e.g., Asperger’s syndrome), currently receiving methylphenidate, or rejects providing informed consent.

An exit criterion is transition to psychosis. Participants who convert to psychosis will be assessed with the 12-month assessment battery.

### Interventions

#### Cognitive remediation (CR) in the experimental group

The CR is conducted in a group setting using both computerised exercises and group exercises. Patients assigned to the experimental intervention group will receive 2 h of CR (1 h of neurocognitive training and 1 h of social cognitive training) once a week for a total of 24 weeks. The training is done in an open group format. In addition to the group training there will be a total of 12 individual sessions of about 50 min aiming at maximising the bridging of the cognitive training to the everyday functioning of the patients, as well as working with individual goals.

The CR is delivered by a cognitive specialist in collaboration with a psychology student with knowledge of cognitive psychology. There will be a maximum of eight participants in each group; thus the therapist-to-patient ratio will be 1:4 or less.

If a patient meets the exit criterion “transition to psychosis”, he or she will be excluded from the intervention but still participate in assessments and statistical analyses, in accordance with the intention-to-treat principle.

#### The neurocognitive training

The neurocognitive remediation will be done by using the Neuropsychological Educational Approach to Cognitive Remediation (NEAR) [46,47]. NEAR is an evidence-based approach that focuses on aspects such as learning and motivation when doing CR. The patients will receive individual neurocognitive training on a computer followed by a group discussion that aims at relating the cognitive exercises to real-world activities. In addition to the group training the patients are instructed to do at least 1 h per week of computerised training at home.

#### The social cognitive training

The overall aim of the social cognitive training is to enhance the skills that enable the patients to understand the thoughts and intentions of others and to respond adequately in social situations. The social cognitive training will be by use of the Social Cognition and Interaction Training (SCIT) manual developed by Roberts et al. [48], which takes the form of group psychotherapy and skills training. It addresses several of the key social cognitive domains, comprising intolerance of ambiguity, attributional biases in explaining negative events, theory of mind (ToM), and emotion perception abnormalities*.* It is the assumption that these social cognitive skills hinder adequate social behaviour in schizophrenia and psychosis-like states.

#### Individual sessions

The individual sessions serve the purpose of maximising the transfer of the effect of the group training to the daily lives of the patients. The individual sessions are embedded in cognitive behavioural therapy (CBT) and use core CBT techniques such as goal setting, modifying distorted thinking, problem solving, and role-play. The content of the individual sessions will be tailored to fit the specific problems of the patient. The sessions will follow a manual that frames the sessions. However, it must be taken into account that the sessions cannot be completely manualised as the therapist approach and CBT techniques used will depend on the individual problem.

### Fidelity to treatment manual

The intervention is manual-based, which improves standardisation of the treatment. David Roberts, co-author of the SCIT manual [48], has given a training course at the Mental Health Centre Copenhagen to ensure a proper understanding and use of the SCIT manual as well as adherence to it. The therapists will be offered bi-weekly Skype supervision from Dr Roberts throughout the trial.

A selected number of group sessions will be videotaped and used to rate adherence to the treatment manual by independent raters. The adherence will be rated by use of the SCIT Fidelity Scale (appendix in the SCIT manual) [48].

### Standard intervention in the experimental group

The standard intervention in the experimental group is planned to be similar to the standard intervention in the control group (see description below).

### Standard interventions in the control group

Patients allocated to the control group are free to choose whatever standard treatment they are offered by the clinicians managing their treatment. Usually standard treatment consists of a somewhat regular contact to health professionals in the in- and outpatient facilities in the capital region of Denmark (e.g., community psychiatric centres or private specialists in psychiatry). It involves monitoring of psychopharmacological treatment and different kinds of supportive counselling, e.g., concerning their psychiatric symptoms, relating to functional domains, or a more regular psychotherapeutic intervention depending on the nature of the health-service managing their treatment. In contrast to the intervention group, standard treatment does not involve specific cognitive training.

The standard treatment provided to both the experimental and control group will be carefully registered in retrospect at the end of the trial by an independent blinded assessor using a checklist comprising items such as use of medication, psychological interventions, number of sessions, and therapist skills.

#### Medications

Patients in both treatment groups are allowed to receive medication. In case an antipsychotic-naïve patient develops psychosis during the trial, this will be reported to the health service managing the treatment in order for them to initiate antipsychotic treatment.

### Assessments

Assessments will be conducted at baseline, prior to randomisation, as information from the baseline assessment is used to perform stratified randomisation and validate inclusion and exclusion criteria. The assessments will be performed at cessation of treatment 6 months after randomisation and at follow-up 12 months after randomisation.

#### Diagnosis

The Comprehensive Assessment of At-Risk Mental States (CAARMS) [45] is used to classify patients as being at UHR for psychosis as well as assessing for transition to psychosis during the trial period. The Structured Clinical Interview for DSM-IV (SCID) [49,50] will be used to assess diagnosis.

The CAARMS is a validated instrument showing good to excellent reliability [45].

#### Neurocognitive function

Neurocognitive function will be assessed using a comprehensive test battery including the Danish Adult Reading Test (DART), a Danish adaptation of the National Adult Reading Test [51]; four subtests from the Wechsler Adult Intelligence Scale-Third Edition (WAIS-III) [51]; Vocabulary, Similarities, Block Design and Matrix Reasoning; the Brief Assessment of Cognition in Schizophrenia (BACS) battery [52]; and the Behavior Rating Inventory of Executive Functions-Adult Version (BRIEF-A) [53]. Furthermore, patients will undergo the following computerised tests from the Cambridge Neuropsychological Test Automated Battery (CANTAB) [51]: Motor Screening Test, Spatial Span, Spatial Working Memory, Emotion Recognition Task, Stockings of Cambridge, IED Set Shifting Test, Paired Associate Learning, 5-Choice Serial Reaction Time, and Rapid Visual Information Processing.

The BACS is specifically designed to detect cognitive changes in response to treatment. The validity and reliability properties of the BACS have been established in patients with schizophrenia and healthy controls, and the BACS composite score has proven high test-retest reliability, increasing the likelihood of detecting a treatment-related effect [52,54,55].

The tests included in the CANTAB battery have proven high validity [56,57]. The reliability of the CANTAB tests varies between individual tests. High reliability (*r* > 0.8) has been shown on measures of visual processing, e.g., the Paired Associates Learning task, while lower reliability has been found particularly on measures of executive functions, e.g., sub-measures of the IED Set Shifting Task [58]. This lower reliability of tests that assess executive functions is very difficult to avoid because of the necessary task novelty involved in assessing executive processing [58].

#### Social cognitive function

Social cognitive function will be measured with The Awareness of Social Inference Test (TASIT) [59], the High-Risk Social Challenge task (HiSoC) [60], the Social Cognition Screening Questionnaire (SCSQ) [61], and Social Responsiveness Scale (SRS) [62].

#### Symptomatology

General symptomatology, negative, depressive, and manic symptomatology will be measured with the Brief Psychiatric Rating Scale Expanded Version (BPRS-E) [63], the Scale for the Assessment of Negative Symptoms (SANS) [64,65], the Montgomery-Åsberg Depression Rating Scale (MADRS) [66], the Young Mania Rating Scale (YMRS) [67], and the Clinical Global Impression (CGI) [68]. Nine cognitive-perceptive basic symptoms from the Schizophrenia Prediction/Proneness Instrument-Adult Version (SPI-A) [69] will be used as a measure of subjectively experienced cognitive-perceptive symptoms.

#### Psychosocial function

Three measures will be included to assess psychosocial functioning: the Personal and Social Performance Scale (PSP) [70], Social and Occupational Functioning Assessment Scale (SOFAS) [71], and Global Functioning: Social and Role Scales [72].

#### Other assessment tools

Five other assessment tools will be used assessing family history, premorbid functioning, substance use, and quality of life. An abbreviated version of the Family Interview for Genetic Studies (FIGS) Family History Index (FHI) (Maxwell M.E: Family interview for genetic studies (FIGS); Manual for FIGS. Unpublished manuscript) is used to assess family history of psychiatric disorder, the Premorbid Adjustment Scale (PAS) [73] assessing premorbid functioning level, Alcohol Smoking & Substance Involvement Screening Test (ASSIST) (WHO ASSIST working group 2002 [74]) assessing substance use, and Quality of Life Scale (QOLS) [75] assessing the patients’ perceived quality of life. At cessation of treatment after 6 months the patients’ satisfaction with the received treatment will be evaluated using the Client Satisfaction Questionnaire (CSQ) [76].

### Adverse events

The participants’ well-being will be a primary focus during the trial. Accordingly, their safety will be monitored and accurately reported throughout the treatment period. CR is not known or expected to cause adverse events [77]; therefore, we do not expect any adverse events to occur. However, should an adverse event occur, it will be dealt with properly by the therapists in charge of the treatment. Using our outcome instruments we will investigate whether the FOCUS intervention causes nominally worse scores, which will be interpreted as a possible indication of harm.

### Setting of assessment

All the assessments will take place at the Mental Health Centre Copenhagen. Most tests are assessed using paper and pencil with the exception of CANTAB, which is a computerised test battery, and the HiSoC test, which uses videotaping. The staff members performing the SCID interview are psychologists and medical doctors who have undergone a 4-day training course using the SCID diagnostic interview co-supervised by Dr Joseph Ventura.

### Data management

All the data will be stored in locked cabinets at the Mental Health Centre Copenhagen in pseudo-anonymised form (i.e., identifiable only with project codes, which are stored separately from the project key identifying the codes). The data will be completely anonymised after publication of all results. The participants’ privacy will be protected by the Danish Data Protection Agency, which has approved the trial.

Databases will be kept locally at the Mental Health Centre Copenhagen under blinded conditions (see Blinding).

### Outcomes and sample size calculation

#### Primary outcome

The primary outcome is overall cognitive function, measured with the BACS composite score 6 months after randomisation.

### Sample size calculation

The sample size calculation is based on our primary outcome, a priori defined to be the between-group difference at 6 months on the BACS composite score. We consider a clinically relevant difference on this scale to correspond to a Cohen’s *d* of 0.50 (e.g., assuming a between-group difference of 3.0 and a pooled SD of 6.0) [29]. With a two-sided alpha of 0.05 and power of 80%, this will require 63 participants to be randomised to each of the two interventions.

### Secondary outcomes and power calculations

The difference in global personal and social functioning 6 months after inclusion will be measured with the PSP. With 63 participants in each group, and assuming a pooled standard deviation of 10 points [72,78-81], we will have more than 97% power to detect a difference of 7 points between the groups, which would be considered the minimal clinically relevant difference on this scale [82].

The BPRS-E will be used to measure symptomatology (e.g., psychosis-like symptoms). The BPRS scale is expected to have a pooled standard deviation of 20.0 points [83]. Given a sample size of 63 per group and a two-sided alpha of 0.05, we have 80% power to detect a difference between the two groups of at least 10.06 points. Antipsychotic treatment and psychotherapeutic interventions have been shown to reduce BPRS by more than 12 points [5,84], indicating that we have sufficient power to detect the minimally relevant difference on this secondary outcome.

Negative symptomatology will be assessed using the SANS. The SANS scale is expected to have a pooled standard deviation of 13 points [5]. Given a sample size of 63 per group and a two-sided alpha of 0.05, we have 80% power to detect a difference between the two groups of at least 6.54 points. Treatments with cognitive and supportive therapy as well as risperidone have been shown to reduce SANS by more than 7 points [5]. This indicates that we have sufficient power to detect the minimally relevant difference of this secondary outcome.

Depressive symptoms will be assessed using the MADRS. Data from a previous randomised clinical trial with UHR patients shows a SD of 9.56 points [6]. Allowing for larger variance in data we set a SD of 10 when calculating detectable differences. With our sample size of 63 per group and a two-sided alpha of 0.05, we have 80% power to detect a difference between the two groups of at least 5.03 points.

### Exploratory outcomes

Exploratory outcomes will be transition to psychosis assessed by the CAARMS; symptomatology assessed by the CAARMS and SPI-A; and social cognition assessed by the ERT, TASIT, HiSoC, SCSQ, and SRS. Psychosocial function will be measured with the SOFAS and Global Functioning: Social and Role Scales. Additionally, the BRIEF-A will be used as a proxy measure of daily functioning. Patients perceived quality of life will be assessed with the QOLS, and lastly the number of participants experiencing adverse events will be recorded.

### Randomisation

Randomisation will be centralised with a concealed randomisation sequence carried out by the Copenhagen Trial Unit (CTU). Randomisation will be stratified by current use of antipsychotic medication (yes/no) and the IQ score (≤100/>100). Block size will be unknown to the investigators and clinicians.

Signed informed consent will be obtained prior to randomisation. The randomisation is computerised and central. The blinded assessors will enter the participant’s data on a webpage hosted by the CTU. Thereafter, a computerised randomisation is performed, and an email is sent to an independent member at Mental Health Centre Copenhagen revealing to which intervention programme the participant has been allocated. The staff member then contacts the participant and informs him or her of the result of the randomisation. The randomised intervention allocation is concealed until the statistical analyses of resulting data have been completed.

### Blinding

The patients and treatment providers will not be blinded. The blinding applies to researchers involved in assessments, data management, data analysis, and drawing outcome conclusions. For the follow-up interviews the patient is instructed in advance not to reveal what type of treatment was received.

### Statistical analyses

The planned comparisons between the two groups on continuous outcomes will be carried out with a generalised linear model adjusted for stratification variables, potential baseline imbalances, and skewed attrition, with missing data handled by multiple imputations. As an important secondary assessment of this type of outcome, linear mixed model analyses with repeated measurements and an unstructured covariance matrix will assess the interaction term between time and intervention. For non-normally distributed continuous outcome measures, non-parametric analyses will be applied. For dichotomous outcomes, logistic regression will be applied, and for time to transition, Cox proportional hazards regression will be applied. All analyses will be according to the intention-to-treat principle, analysing all participants in the groups they were assigned to by randomisation. We expect to encounter missing data, and this will be handled with the linear mixed models and multiple imputations as appropriate. A blinded and independent statistician who has no contact to the trial participants will conduct the primary efficacy analyses.

### Ethical consideration

The trial has obtained approval by the Regional Ethics Committee of Zealand (H-6-2013-015) and the Danish Data Protection Agency (RHP-2014-009-02670). The trial is registered at ClinicalTrial.gov as NCT 02098408. Positive as well as neutral and negative results of the trial will be published in international journals.

The participants will receive information on the trial both verbally and in written form. Written informed consent will be obtained from each participant before inclusion in the trial. It is emphasised that participation in the trial is voluntary and that the participant can withdraw his or her consent at any time without consequences for treatment possibilities. We will upload depersonalised individual patient data to be used in meta-analyses on Zenodo via OpenAIRE (https://www.openaire.eu/).

## Discussion

There are several strengths in the design of the FOCUS trial. First, to our knowledge no other randomised clinical trial has assessed the effect of both neurocognitive and social cognitive remediation in a UHR population. Second, the CR has a primary focus on linking cognitive remediation to real-life improvements. Third, it is a large-scale trial. As UHR patients are difficult to attain, the sample size of 126 UHR patients makes it one of the biggest UHR intervention trials to date. Fourth, it employs observer-blinded assessment of outcomes with the intention to ensure that the outcome data are assessed without bias [85]. Moreover, data management, data analyses, and conclusions will be conducted and drawn blind to the intervention group. Fifth, assessing outcome at cessation of treatment after 6 months will allow evaluating the immediate effect of the CR, whereas the 12-month follow-up evaluates the long-term effects of the CR intervention. Sixth, the wide range of outcome estimates in the trial allows the opportunity to assess outcome in multiple areas of cognition, symptomatology, and adaptive functioning.

The trial may have some limitations as to the best of our knowledge no published trial has investigated the effect of combining the SCIT treatment with a neurocognitive remediation programme. Hence, we lack knowledge on the feasibility of this approach. However, as stated in the Background section, evidence shows that both the neurocognitive and social cognitive domains are impaired in UHR patients. Therefore, it seems essential to try to target both domains. This is further supported by the hypothesis that social cognition acts as a mediator between neurocognition and real-world outcome. Furthermore, it seems highly effective to combine treatment modalities in CR [29,86]. Another limitation is that the experimental intervention is an add-on to standard treatment. This design could potentially cause the problem of patients in the intervention group receiving less standard treatment as a result of participating in the trial.

## Trial status

Trial initiation was April 2014. By November 2014, 19 patients had been randomised.

## Abbreviations

CR: Cognitive remediation
CTU: Copenhagen trial unit
SCIT: Social cognition and interaction training
RCT: Randomised clinical trial
ToM: Theory of mind
UHR: Ultra-high risk
